# Pathogen Control in the Built Environment: A Probiotic-Based System as a Remedy for the Spread of Antibiotic Resistance

**DOI:** 10.3390/microorganisms10020225

**Published:** 2022-01-20

**Authors:** Maria D’Accolti, Irene Soffritti, Francesca Bini, Eleonora Mazziga, Sante Mazzacane, Elisabetta Caselli

**Affiliations:** 1 Department of Chemical, Pharmaceutical and Agricultural Sciences and LTTA, University of Ferrara, 44121 Ferrara, Italy; maria.daccolti@unife.it (M.D.); irene.soffritti@unife.it (I.S.); francesca01.bini@edu.unife.it (F.B.); eleonora.mazziga@edu.unife.it (E.M.); 2 CIAS Research Center, University of Ferrara, Via Saragat 13, 44122 Ferrara, Italy; sante.mazzacane@unife.it

**Keywords:** built environment, microbiome, sanitation, probiotics

## Abstract

The high and sometimes inappropriate use of disinfectants and antibiotics has led to alarming levels of Antimicrobial Resistance (AMR) and to high water and hearth pollution, which today represent major threats for public health. Furthermore, the current SARS-CoV-2 pandemic has deeply influenced our sanitization habits, imposing the massive use of chemical disinfectants potentially exacerbating both concerns. Moreover, super-sanitation can profoundly influence the environmental microbiome, potentially resulting counterproductive when trying to stably eliminate pathogens. Instead, environmentally friendly procedures based on microbiome balance principles, similar to what applied to living organisms, may be more effective, and probiotic-based eco-friendly sanitation has been consistently reported to provide stable reduction of both pathogens and AMR in treated-environments, compared to chemical disinfectants. Here, we summarize the results of the studies performed in healthcare settings, suggesting that such an approach may be applied successfully also to non-healthcare environments, including the domestic ones, based on its effectiveness, safety, and negligible environmental impact.

## 1. Introduction

**Figure 1:**
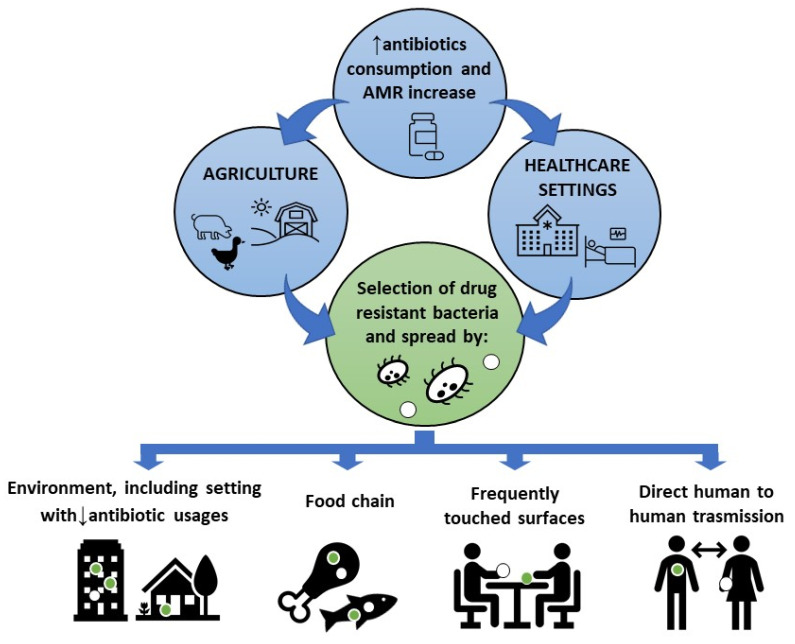
AMR spread from high antibiotic usage areas to settings where antibiotic usage is significantly lower, via different ways of transmission.

Environmental pollution and antimicrobial resistance (AMR) are major public health challenges of our time. According to the European Centre Disease and Control (ECDC), each year in Europe at least 4 million people catch an antibiotic resistance infection, leading to over 40,000 deaths [1], and WHO estimated at least 10 million deaths within 2050, if no concrete global action is taken against AMR as soon as possible [2]. AMR has primarily developed as a result of the selective pressure exerted by the widespread use of disinfectants and antibiotics in the hospital settings, as well as in agriculture [3], livestock, and in the general community. As a consequence, the microorganisms persistently contaminating those environments became drug, multi-drug, or pan-drug resistant (MDR, panDR), then spreading rapidly through the environment (including that with low antibiotic usage such as private homes), the food chain, the frequently touched surfaces, or via direct contact between humans (Figure 1).

Consistent with this, several countries have introduced and developed policies to reduce the use of antibiotics both in the healthcare and non-healthcare settings, applying the so-called “One health” approach [4], and supporting surveillance programs and improvement of environmental hygiene to prevent the spread of MDR microbes [5,6]. Of note, the emergency management of the current pandemic has mandatorily imposed a massive use of chemical disinfectants in both the healthcare and non-healthcare environments, in the attempt to prevent SARS-CoV-2 transmission, and this has impacted negatively on both the environmental pollution and the AMR spread, as reportedly evidenced [3,7]. Disinfectants may in fact not only induce direct resistance towards disinfectant compounds [8], but also cross-resistance towards antibiotics. For example, Gram-negative species adapted to benzalkonium chloride can become resistant to ampicillin, cefotaxime, and sulfamethoxazole [7]. Chlorhexidine can favor resistance to ceftazidime, sulfamethoxazole, imipenem, cefotaxime, and tetracycline, as well as against colistin. Cross-resistance to antibiotics was also found with triclosan, octenidine, sodium hypochlorite, and didecyldimethylammonium chloride [3,7]. Consistent with this further risk of AMR diffusion, the WHO feared a risk for a future AMR pandemic as a consequence of the anti-COVID-19 measures [9]. The COVID-19 pandemic has in fact deeply influenced our perception of hygiene and sanitation procedures, and the guidelines introduced by regulatory bodies for COVID-19 control in healthcare and non-healthcare settings indicate the use of high-level disinfectants, including sodium hypochlorite, hydrogen peroxide, and alcohol, for sanitization [10,11]. Indeed, controversies exist about the need of disinfectants instead of cleansers, especially in low-risk healthcare or in non-healthcare environments [11,12], as the excessive use of disinfectants may represent a threat for people [13] and increase water and hearth pollution [14]. Moreover, despite their immediate effectiveness, disinfectants have a temporary effect, as they are not able to prevent recontamination phenomena that occur continuously [15,16] and can favor AMR. Of note, more and more infections sustained by MDR microbes occur in the general population [17], although the incidence is lower compared to that recorded in the hospital, indicating that pathogen contamination and AMR are not concerns limited to healthcare settings but they are spreading even in domestic environments. The AMR onset and spread in the population is influenced by microbial, host, and environmental factors, including exposure to antimicrobials in clinical medicine, agriculture, livestock, food production, and environmental contamination [18,19,20]. Several non-healthcare settings including public transports, schools, universities, and other highly frequented environments can become reservoirs for AMR spread, including in private homes. Indeed, for many years cleaning alone has been proposed and used effectively for hygienization purposes in the hospital environment [21], suggesting that the massive use of high-level disinfectants in the community or domestic environments may not be needed. This also in light of the SARS-CoV-2 susceptibility to common detergents, such as sodium laureth sulphate [22], and of the recognized primary role of hand hygiene, besides environmental sanitation, in fighting AMR and preventing infection transmission [23].

## 2. The Built Environment Microbiome

Indoor spaces and built environments (BE) have become the main habitat for individuals, including homes, workplaces, schools, public transports, and hospitals [24]. Human beings spend there most of their time and are consequently continuously exposed to indoor chemical and biological factors, which can ultimately profoundly affect their lives and health [25]. Recently, the community of microorganisms inhabiting the built environment has gained much consideration as an important component of indoor exposures potentially having a primary role in the risk to develop human diseases [26]. Similar to living organisms, BEs can be considered super-organisms with their own microbiome, which can include bacteria, viruses, and fungi, inhabiting inanimate or organic surfaces, where microbes can persist for long periods, potentially being transmitted to other individuals [27,28,29]. A “healthy” BE microbiome is predominantly composed of commensal and beneficial microbes spread by humans and pets, but it can also contain pathogens potentially responsible for human diseases, spread by colonized or infected individuals and/or selected by disinfectant and antibiotic usage. Besides surfaces, the air can also be a reservoir for potentially pathogenic microbes, especially in indoor environments equipped with air ventilation systems [30]. Consistent with this, the Sick Building Syndrome (SBS) is defined as a pathological condition that can be developed as a consequence of living in a contaminated environment and breathing unhealthy air contaminated by microbes including bacteria, fungal spores, and molds, which can proliferate favored by specific humidity and temperature conditions, and can be transported by air ventilation [31].

The BE microbiome is not static, as it is part of a dynamic and complex ecosystem characterized by several organisms that interact each other and with the surrounding environment [25], in a continuous interplay between the building itself and occupants [32]. Recent studies began to characterize the BE microbiome, showing that it can include several variable taxonomic groups [5], with different types of indoor spaces exhibiting different microbiome structure, abundance and diversity [33,34,35]. The factors influencing the BE microbiome include geographical and seasonal variations, as well as by abiotic, biotic, and anthropogenic factors (i.e., human activities and occupancy) [36]. Humans influence the BE microbiome essentially by spreading their own microbes in the occupied indoor environment (with higher levels of occupancy corresponding to higher abundance of microbes), and by moving the indoor particles through their movements, which can cause the resuspension of the settled particles [26].

Several studies aimed to define the BE microbiome to assess the risk and mode of transmission of pathogens between built occupants, and recent metagenomics technologies have highlighted that more confined BE have less microbiome biodiversity and more resistant (MDR) species [32,37,38]. Overall, the areas where a high selective pressure is exerted by the massive use of disinfectants/antimicrobial display the highest rates of MDR microbes (including hospitals but also in animal husbandry and agriculture) [39]. However, resistant bacteria can also be detected and transmitted by other indoor environments, including domestic ones [40,41,42], although the latter generally show lower AMR compared to hospitals.

Interestingly, instead, increased microbial diversity was suggested to be associated with prevention of allergy and asthma, and children growing up on a farm have reduced risk of inflammatory respiratory diseases compared to children raised in more urban environments [43,44]. Consistently, up to 25% of the human microbiome variability appears related to the environmental input, rather than ethnicity [45]. Drugs and disinfectants can obviously affect the microbiome in a profound way, and an increase in allergic and chronic inflammatory diseases was observed in Western countries in contrast with the low prevalence in low-income countries [46,47,48], likely consistent with the reduced environmental microbial stimulation correlated to increased sanitation, which is consistent with the “hygiene hypothesis” [49,50,51]. Reduced biodiversity of the environmental microbiome from soil and air has also been implicated in adverse health outcomes, and given the substantial importance of the microbiome environments on health, especially in shaping the microbiome in the early childhood, it is surprising that there are sparse studies investigating this aspect [52].

This concept was recently summarized in the “Holobiont” concept, highlighting that the urban biodiversity is associated with the human health, and that the industrial urbanization might disrupt the symbiosis between microbiota and its host, leading to negative health outcomes [53]. These observations also suggest that restoring urban microbial biodiversity and micro-ecological processes through microbiome rebalancing could lead to obtain holobiont health and prevent the development of diseases associated with urban microbiome.

As mentioned, conventional chemical cleaning shows important limitations, first and foremost the selection of MDR microbes [3,7], together with the temporary action and important environmental impact [13,16]. Similar to what is considered for human beings, where the depletion of commensals is associated with loss of microbial biodiversity and increased risk of disease, whereas restoration of biodiversity enhances health-associated conditions [54], the addition of beneficial microbes may instead enrich the BE microbial community rendering it safer and healthier.

## 3. Pathogen Diversity in the Built Environment Microbiome

The BE microbiome composition is largely sourced from humans and commonly include high amounts of human skin colonizers such as Gram-positive Staphylococci, but also Gram-negative bacteria including Enterobacteriaceae family, fungi, and viruses. In particular, data from the Home Microbiome Project showed strict relationships between microbes, people and their homes, suggesting rapid colonization of the home environment by the family’s microbiota [55,56]. Various bacterial species can reside in the toilet but also in the kitchen and refrigerators, potentially being a direct source of food borne illness [57]. Proteobacteria, Firmicutes, Actinobacteria, and Bacteroidetes were detected in private homes, including *Propionibacterium*, *Bacteroides*, and *Staphylococcus* genera [57]. *Staphylococcus* and *Micrococcus* genera were also detected in washing machines, half of which potential opportunistic pathogens, emphasizing the need for effective cleaning and control strategies [58].

### 3.1. Staphylococcus Species

Staphylococci, usual colonizers of the human skin and upper respiratory tract, are commonly spread from humans to BE surfaces and air [59,60,61]. Many staphylococcal species can become opportunistic pathogens and cause severe diseases including blood-stream, lung, soft tissue, and skin infections [62], but also atopic diseases such as asthma and hay fever [63]. Urbanization and domestic environments were reported to play an important role in the development of chronic conditions and allergic diseases associated with *Staphylococcus* spp. [64], which are known to persist long in domestic dry environments and surfaces [65,66]. Among *Staphylococcus* species, *Staphylococcus aureus* can cause community- and hospital-associated infections [67,68], and its increasing AMR during the last decades renders it one of the major agents of nosocomial and community-associated infections [69]. Originally detected only in the healthcare settings, Methicillin resistant *S. aureus* (MRSA) has now spread also to domestic environments [70]. Similarly, coagulase-negative staphylococci (including *S. epidermidis*, *S. haemolyticus*, *S. hominis* and *S. saprophyticus*, all associated with severe infections in the hospital setting) are now often found also in non-healthcare settings [71], which play an important role in horizontal transfer of AMR genes [72].

### 3.2. Enterobacteriaceae

Enterobacteriaceae, a large family of Gram-negative bacteria commonly present in the human gut microbiota, can also be found in food, water, and home surfaces [73,74]. Among such family, the gut commensal *E. coli* can cause gastroenteritis, urinary tract infections, respiratory illness, and pneumonia [75]. Of note, bacteria belonging to the Enterobacteriaceae group show widespread beta-lactam resistance and carbapenem resistance [76], and have been also often reported in community non-healthcare environments, highlighting the potential threat for public health [77,78]. Furthermore, the recent appearance and spread of mobile colistin resistance (*mcr*) genes among MDR Enterobacteriaceae rendered inefficient even the last-resort colistin drug [79], with further important consequences for human health [80].

### 3.3. Fungi

The indoor mycobiome is largely composed by saprotrophs able to degrade indoor available organic substrates, including *Cladosporium*, *Aspergillus, Penicillum* [81,82]. Its diversity, abundance, and composition are determined by factors including climate, but also by local environmental variations including construction features and building functions, which are the products of dynamic interactions [26,83]. In moist environments, fungal growth with subsequent release of spores, hyphal fragments, and mycotoxins can act as a source of indoor pollution [84], and the consequent poor indoor air quality is associated with diseases including asthma, allergies, and other respiratory pathologies [85].

Like other contaminant microorganisms, fungi can survive for days to months on BE surfaces [29], interacting each other or with bacteria. Dynamic fungal–bacteria interactions may play important roles in disease occurrence [86], since some bacteria produce compounds that enhance fungal virulence, whereas others produce antimicrobial factors that inhibit pathogenesis by repressing hyphal growth [87]. Consistent with this, the artificial introduction of apathogenic bacteria able to counteract fungal growth and virulence may potentially decrease the risk of developing mycoses. Probiotic *Bacillus* spp., for example, are considered good fungal competitors [86], and are consistently used as fungicides in agriculture [88]. In particular, *B. subtilis* and *B. pumilus* spores (commercially available in the Serenade and Sonata products; Bayer) are used to inoculate the soil and prevent fungal diseases in plants [89], showing an effective fungicide action after germination into the vegetative bacterial form.

### 3.4. Viruses

Compared to the knowledge on bacteria and fungi of the BE microbiome, less information is available about the viral community, essentially provided by the recent advance in metagenomics analyses [90]. The main sources of viruses are represented by humans, pets, plants, indoor ventilation and air-conditioning systems, and dust [30]. Metagenomics studies evidenced both beneficial viruses (including animal and bacterial viruses such as the bacteriophages), and potentially pathogenic viruses, including respiratory viruses [91,92,93]. In particular, respiratory viruses, including also the new SARS-CoV-2 human coronavirus, are mainly spread through respiratory droplets [94]; thus, the indoor spaces are a primary context in which such transmission occurs, both by direct human-to-human interaction and through the air [95,96,97]. Recent data also highlight the potential role of contaminated surfaces and fomites as a possible transmission route for SARS-CoV-2 [98,99,100], based on its ability to survive up to days on inanimate surfaces [101], similarly to other enveloped viruses including influenza viruses and herpesviruses [29,102].

## 4. A New Paradigm of the Environmental Health: Focus on Microbiota Remodulation

The recent studies highlighting the continuous relationship between the BE, its microbiome, and occupants, provided a base for designing intervention strategies aimed to restore a healthy BE microbiome through the rebalance of the indoor microbial community. Compared to the healthcare environment, there is little surveillance of AMR in human non-healthcare settings; however, MDR bacteria transmission has been reported in many different public urban settings, where high-density human activity can be detected, such as public transport, sports arenas, and schools [70]. The COVID-19 pandemics has introduced massive chemical disinfection in all BE types, but the AMR threat for public health should not be forgotten while global attention is focused on the COVID-19 pandemic. To overcome the limitations of chemical disinfections, new cleaning strategies have been proposed to counteract pathogens and their AMR, based on the concept of “bidirectional hygiene” (or Bygiene) [103], which consider to replace pathogens with beneficial microbes exploiting the mechanism of competitive exclusion. In humans it is known that a microbiome depletion (for example after a prolonged antibiotic therapy) can favor the colonization of potentially pathogenic microorganisms [104,105]. Similarly, a super-sanitation aimed to eliminate all the microbes from the BE would likely be unsuccessful, instead allowing pathogen colonization and AMR increase [25].

Toward this direction, studies performed by us in the healthcare settings showed that probiotic-based strategies can rebalance the hospital microbiome, leading to stable reduction of pathogen contamination, of its AMR, and of the associated infections [14,106,107,108,109,110]. Such a cleaning system (Probiotic Cleaning Hygiene System, PCHS), consisting of an eco-friendly cleanser with added spores of probiotic bacteria belonging to the *Bacillus* genus, can in fact competitively exclude the re-growth of pathogenic species, including bacteria and fungi [14,109]. In particular, single and multicenter studies in the healthcare setting proved the PCHS effectiveness in abating, around 80% more than chemical disinfection, major nosocomial pathogens, including *Staphylococcus* spp., *Enterobacteriaceae* spp., *Acinetobacter* spp., *Pseudomonas* spp., *Candida* and *Aspergillus* spp., and sporogenic *C. difficile*. Importantly, such a remodulation was stable and did not select resistant strains, rather abating up to 3 Logs (99.9%) the previously present resistances [14,110]. As a consequence of stable microbiome remodulation, the use of PCHS was associated with a 52% reduction of HAI incidence [108], accompanied by a 60% decrease in HAI-related antimicrobial drugs consumption, and by a 79% decrease in the costs associated with the management of infections [107]. PCHS action was also recently tested against viruses, showing its inactivation ability against different enveloped viruses, including SARS-CoV-2 (−99.99% within 1 h) [16]. This suggests that PCHS may be effectively used to reduce the risk of virus transmission via the indoor environment, concomitantly avoiding the further selection and spread of the AMR pandemics.

Since thus far most surveys have been performed in Italy, due to exclusive availability of PCHS in that country, it would be important to obtain data confirmation from other research groups in other countries. However, based on the hitherto collected data, on the PCHS ease of use, and on its eco-sustainable features, we argue that PCHS would have the potential to be effectively used in different BE types, including public and private non-healthcare environments, to stably and gradually restore healthy BE microbiomes. In fact, similar to what was observed in healthcare settings, the domestic microbiome is largely derived from human occupants, and massive use of bactericidal and disinfecting products in the domestic environment could influence the microbiome composition, rendering it more aggressive and resistant. In contrast, the application of competitive exclusion principles may counteract the proliferation of existing pathogenic species and prevent further colonization, stably modulating the microbiome in a more ecological way. In brief, although high level disinfection will continue to be necessary in specific areas (i.e., surgical rooms, sterile rooms, etc.) or against specific biological threats (such as the presence of highly resistant and hazardous pathogens belonging to the class of risk 3 or 4), detersive cleaning with less pollutant systems may provide adequate protection and prevention from infectious risk, especially in the community or domestic environment.

**Figure 2:**
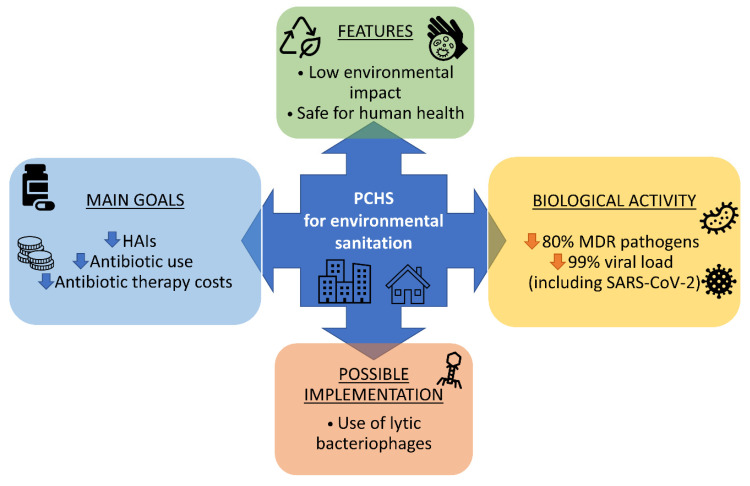
Schematic representation of PCHS features, biological targets, main experimental goals, and potential implementation.

As a further potential improvement, at the moment limited to the hospital environment, specific removal of individual bacterial targets could be accomplished by the addition of lytic bacteriophages to cleansers. Bacteriophages are viruses infecting only bacteria with high specificity and rapidity, and their successful applicability has been reported for the decontamination of food and food-processing surfaces in industrial settings [111]. No studies on the effectiveness of phages in domestic environments are available yet, however their effective application against MDR bacterial strains was reported in the hospital environment [112,113], suggesting that a combined phage and probiotic-based cleaning may provide an effective way to stabilize the BE microbiome in different settings. Figure 2 summarizes the main PCHS features, as well as its biological target and the main goals achieved by the experiments.

## 5. Conclusions

The BE microbiome is diverse and dynamic, and understanding the indoor microbiome pave the way to increased opportunities to make actionable recommendations, which may result from melting microbial-, building practitioner- and health-related data. The health of holobionts, including humans, is fundamentally linked to the health of ecosystems and potentially driven by the state of environmental microbiota.

Since the human microbiome is shaped mostly under the influence of the environmental one, and based on the promising results obtained by using the PCHS system in healthcare settings, we suggest that applying the microbiome balance principles to the community BE may provide an alternative eco-friendly, low-risk, and low-cost hygienization, to rebuild a safe domestic microbiome with potential significant effects on the human health.

In short, our outer world impacts our inner microbial world and vice versa. A beneficial microbial environment may help to protect BE occupants from disease, if it is not bleached to death, and as the research reveals more and more about the relationships between humans and the BE microbiome, understanding and using safe cleaning systems becomes increasingly important.
